# Factors associated with longitudinal hydroxyurea adherence trajectories among individuals with sickle cell disease in Texas Medicaid: A group-based trajectory modeling study

**DOI:** 10.1177/20406207261481884

**Published:** 2026-08-24

**Authors:** Blessing I. Okoye, Godwin Okoye, Jamie C. Barner

**Affiliations:** 1 Health Outcomes Division, College of Pharmacy, 12330 The University of Texas at Austin, Austin, TX, USA

**Keywords:** adherence, group-based trajectory modeling, hydroxyurea, sickle cell disease

## Abstract

**Background:**

Hydroxyurea, first-line therapy in sickle cell disease (SCD), has suboptimal adherence ranging from 10%-36%. Thus, examining longitudinal adherence trajectories may help identify critical timepoints and individual subgroups for targeted interventions.

**Objectives:**

To identify distinct longitudinal hydroxyurea adherence trajectories among individuals with SCD enrolled in Texas Medicaid and to examine sociodemographic and clinical factors associated with trajectory group membership.

**Design:**

Retrospective, longitudinal cohort study.

**Methods:**

A retrospective analysis of Texas Medicaid data (01/01/2016-08/31/2023) included individuals with ≥3 medical claims for SCD, ≥1 prescription claim for hydroxyurea without a claim in the pre-index period, aged 2-63 years on the index date (first hydroxyurea claim), and continuously enrolled in Texas Medicaid for 6 months pre- and 12 months post-index. Proportion of days covered (PDC) was used to estimate adherence. Group-based trajectory modeling (GBTM) was used to identify hydroxyurea adherence trajectories. Multinomial logistic regression was used to examine factors associated with hydroxyurea adherence trajectory group membership.

**Results:**

Of included individuals (N=1,246; mean age=18.1±12.7, 50.8% female), GBTM revealed five membership groups: consistent adherence (16.1%; mean PDC=89.2±8.5%), fluctuating adherence (28.7%; mean PDC=52.6±12.8), sharp decline and fluctuating adherence (16.5%; mean PDC=26.2±8.8), rapidly decreasing adherence (22.2%; mean PDC=25.6±10.3), and early and consistent nonadherence (16.5%; mean PDC=8.4±1.3). Potential intervention timepoints were identified at months 2, 3, and 5, when adherence declined markedly. Age groups 16-21, 22-34, and ≥35 years (reference: 2-15 years), and an increasing number of vaso-occlusive crises (VOC) in the pre-index period were significantly associated with higher odds of being in the less-adherent trajectory groups compared to the consistent adherence trajectory group: adjusted odds ratio (aORs) range: age groups=2.2-8.7; VOC=1.2-1.4.

**Conclusion:**

Unique adherence trajectory patterns with critical timepoints for intervention were observed. Individualized and timely interventions should be tailored to adults, especially those who are transitioning to adult care (22-34 years), and those experiencing frequent VOC.

## Plain language summary

Sickle cell disease (SCD) is an inherited blood disorder that mainly affects Black/African Americans. It causes painful episodes called vaso-occlusive crises (VOC) and other complications that occur over the lifespan. Hydroxyurea is the first-line therapy used to prevent and/or reduce these complications (including pain, stroke, damage to kidneys), but many individuals do not take it consistently. Previous research typically measures adherence as a single average number over time, which does not reveal important changes in how individuals take their medication. This study used group-based trajectory modeling to examine patterns of hydroxyurea adherence among 1,246 individuals with SCD enrolled in Texas Medicaid. Five distinct patterns of hydroxyurea adherence were identified. Only about 16% of individuals with SCD were consistently adherent throughout the year. Others showed different patterns: fluctuating adherence, sharp decline and fluctuating adherence, rapidly decreasing adherence, and about 17% were consistently nonadherent shortly after hydroxyurea initiation. Early declines in hydroxyurea adherence occurred at months 2, 3, and 5. Young adults and adults were much more likely to belong to the lower adherent groups compared to children and adolescents. Individuals with an increasing number of VOC during the baseline period were more likely to belong to the lower adherent groups. These findings suggest that healthcare providers should offer support early, especially within the first few months of treatment. Potential strategies include proactive outreach (e.g., by pharmacists), prescheduled follow-up visits, and educating individuals about the benefits of hydroxyurea and strategies for managing side effects. Extra attention should be given to young adults and adults who may have lapses in consistent care after transition from pediatric to adult care. Individuals experiencing frequent VOC should be educated on the importance of adhering to their medications.

## Introduction

Sickle cell disease (SCD) is a rare inherited hemoglobinopathy caused by a mutation in the hemoglobin-beta gene, leading to the proliferation of dysfunctional hemoglobin-S (HbS).^1^ Approximately 100,000 individuals in the United States (US) are living with SCD, and it disproportionately affects racial/ethnic minority populations, with over 90% of cases occurring in Black/African Americans.^2^ It is characterized by acute and chronic complications, including vaso-occlusive crises (VOC) and progressive organ damage, which contribute to reduced quality of life, morbidity, mortality, reduced life expectancy (54 years), and psychosocial issues.^3–6^ SCD is also associated with frequent healthcare utilization and a lifetime medical cost of $1.7 million (2020 USD).^7,8^

Hydroxyurea has been established as the first-line therapy for individuals with SCD and is recommended by the National Heart, Lung, and Blood Institute.^9^ By increasing fetal hemoglobin and reducing red blood cell sickling, hydroxyurea has been shown to decrease the number of VOC events, acute chest syndrome, hospitalizations, and mortality among individuals with SCD.^10–12^ Despite its established clinical benefits, adherence to hydroxyurea remains suboptimal, ranging from 9.6-35.8% (medication possession ratio (MPR)/proportion of days covered (PDC) ≥80%).^5,10,13,14^ However, most existing studies have estimated adherence to hydroxyurea cross-sectionally using static adherence measures such as PDC and MPR, which provide a summary measure of individuals’ medication-taking behavior over a fixed period. These methods can obscure important variability in adherence over time and provide limited insights into time-varying patterns, given the inherently dynamic nature of medication-taking behavior.^15^

Group-based trajectory modeling (GBTM) is a statistical approach used for characterizing changes in an outcome over time and identifying latent subgroups of individuals who follow similar longitudinal patterns.^16,17^ Unlike traditional adherence measures, GBTM allows for the examination of dynamic adherence patterns and provides insights into heterogeneity that may be obscured by aggregate adherence measures. Hence, GBTM can help identify individuals at higher risk for low adherence and inform the development of targeted interventions. Additionally, identifying distinct adherence trajectories can help clinicians anticipate periods of declining adherence, enabling proactive outreach or counseling before complications arise.^15,16^ Despite the growing use of GBTM to assess medication adherence patterns, its use to assess hydroxyurea adherence remains scarce.^18^ A prior study among children and adults with SCD enrolled in Medi-Cal identified three distinct hydroxyurea adherence trajectories.^18^ Notably, adults with SCD (aged 18-24 and ≥25) had higher odds of membership in the lower adherence trajectory compared with children with SCD (0-17 years). However, this study included only two predictors in its model: age group and the number of prior VOCs that resulted in hospitalization or emergency department utilization, leaving uncertainty about how other important factors may affect longitudinal hydroxyurea adherence trajectories.^18^

To address these gaps, we characterized longitudinal hydroxyurea adherence trajectories using Texas Medicaid data and evaluated a broader set of sociodemographic and clinical characteristics that may be associated with hydroxyurea trajectory group membership. Understanding adherence patterns using GBTM can facilitate the identification of critical time points at which healthcare providers can intervene to discuss treatment goals, reinforce the importance of adherence, manage side effects, and address other concerns that may influence medication adherence among individuals with SCD. Therefore, the objective of this study was to identify distinct longitudinal hydroxyurea adherence trajectories among individuals with SCD enrolled in Texas Medicaid and to examine sociodemographic and clinical factors associated with trajectory group membership.

## Methods

### Study design and data source

This was a retrospective analysis of Texas Medicaid data from 01/01/2016 to 08/31/2023 (study period). The database includes demographic information, inpatient and outpatient claims, and prescription claims. Medicaid is the largest payer for individuals with SCD in the US, covering over 50% of this population. Additionally, Texas has the third-highest SCD prevalence in the US.^19,20^ As such, Texas Medicaid provides a relevant setting to examine longitudinal adherence patterns of hydroxyurea.

### Study population

**Figure 1.**
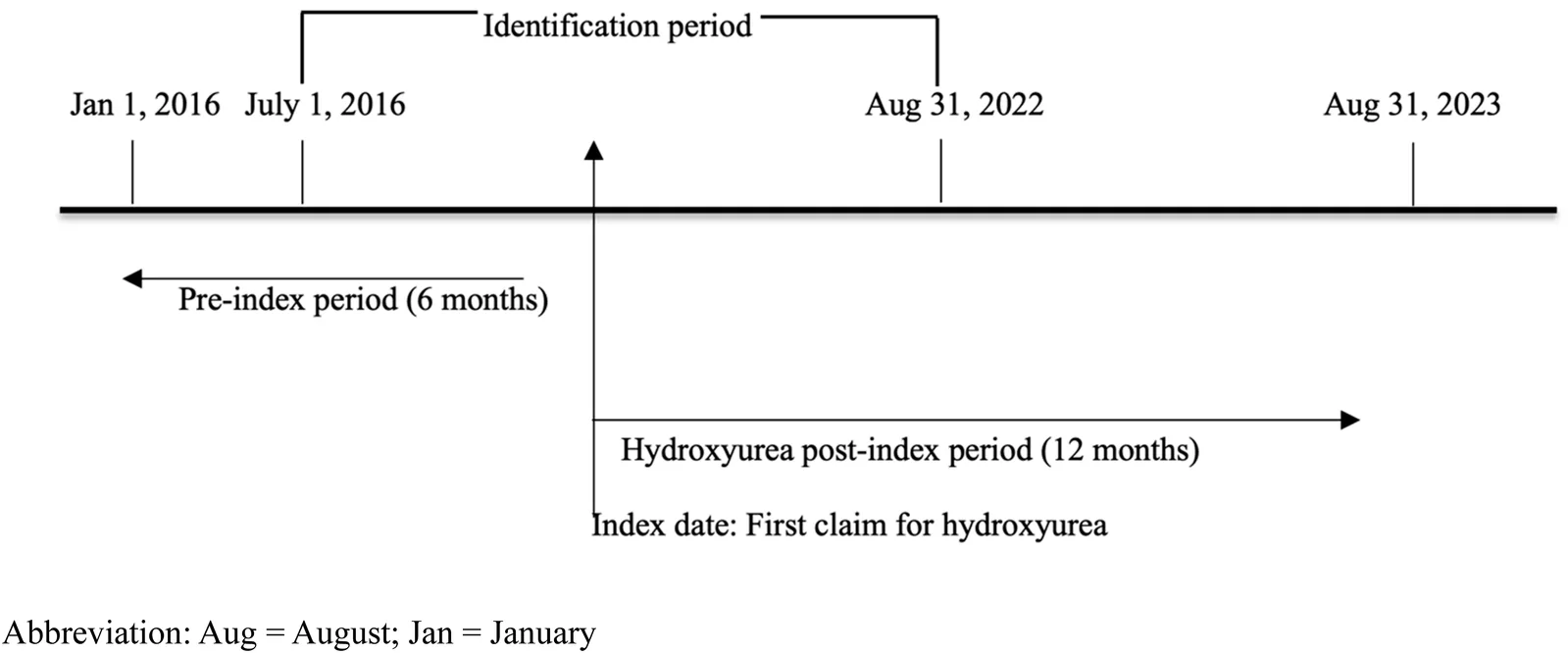
Data extraction and subject identification period. Abbreviation: Aug = August; Jan = January.

To be included in this study, individuals were required to have ≥3 medical (inpatient or outpatient) claims on separate days with International Classification of Diseases (ICD-10) codes for SCD (D57, D57.x (except for 57.3; sickle cell trait), and D57.xx) during the study period^21,22^; ≥1 prescription claim for hydroxyurea during the identification period (07/01/2016 - 08/31/2022) without a prior claim in the 6-month preindex period to implement the new-user design and identify incident hydroxyurea users; aged 2-63 on the index date (first claim for hydroxyurea); and continuous enrollment in Texas Medicaid for at least 6 months before and throughout the 12 months after the index date (Figure 1, N = 1,246). While Rushing et al. examined medication adherence following an initial prescription,^18^ this study used the first prescription fill as the index date. This was done to evaluate adherence trajectories beginning at treatment initiation, thereby capturing early medication-taking behavior following hydroxyurea initiation. Individuals with a diagnosis associated with a non-SCD indication or who received hematopoietic stem cell transplantation (Supplemental Table 1) during the 6 months pre-index were excluded to ensure that hydroxyurea use was attributable to SCD management and to avoid including individuals whose treatment course may have been altered by curative therapy. Hydroxyurea exposure was identified using pharmacy claims and a list of generic code numbers (GCN) corresponding to hydroxyurea and branded formulations available during the study period (e.g., Hydrea^®^, Droxia^®^, and Siklos^®^).

### Study variables

The outcome variable was hydroxyurea adherence trajectory group membership. Adherence was measured using PDC, which is the ratio of the total days covered by a medication based on the days’ supply to the total number of days in an observation period.^23^ Monthly hydroxyurea adherence (continuous) was assessed over 12 months following the index date. In addition, adherence rate (dichotomous), defined as the proportion of individuals with PDC≥80%, was also assessed.

Sociodemographic and clinical characteristics were measured during the 6-month pre-index period and were included as independent variables. Sociodemographic characteristics included age groups, gender, and resource availability. Age group categories are similar to those used in the literature and represent: pediatric care (2-15 years); youth and young adults as they transition to adult care (16-21 years); adults as they are establishing care in adult settings (22-34); and adults who have presumably established a regular source of care (≥35 years).^14,24^ Resource availability was estimated based on the presence of SCD resources (e.g., specialized SCD clinics) within a 50-mile radius of the individual’s residence. The distance in miles was calculated as a straight-line distance between the centroid of an individual’s 5-digit residential zip code and the nearest SCD resource zip code.^24^ Clinical characteristics included: number of SCD-related complications, VOC events, other comorbid medical conditions, and diagnosis of depression and/or anxiety (Supplemental Table 2). Depression and/or anxiety were evaluated separately because they are mental health conditions with well-established influence on medication adherence,^5,25^ whereas SCD-related complications and other comorbid medical conditions reflect physical disease burden.

### Statistical analysis

GBTM with a censored normal distribution was used to identify trajectories of hydroxyurea adherence and estimate the proportion of individuals in each trajectory group. GBTM is a semi-parametric latent class modeling approach that identifies unobserved groups of individuals who follow distinct longitudinal patterns in an outcome over time. GBTM estimates the probability of membership in distinct trajectory groups as a function of observed repeated measures of the outcome over time at the individual level.^16,17^ The model input was continuous PDC for each 30-day period for the 12 months following the index date.

Trajectories were modeled using cubic polynomial functions of time, as they allow for greater flexibility in capturing complex, non-linear adherence patterns over time. The model assumes conditional independence between groups.^17,26^ Using maximum likelihood estimation, individuals were assigned to the trajectory group for which they had the highest posterior probability of membership.^16,17^ Models with 2 to 6 trajectories were estimated using the PROC TRAJ procedure in SAS. Model fit was assessed using 4 criteria: 1) Minimum predicted group proportions; 2) Bayesian information criterion (BIC); 3) Bayes factor; and 4) Clinical interpretability. The first criterion was the predicted group proportion, which was set at a minimum of 10%. Next, BIC was evaluated, with larger values (less negative) indicating better model fit. The Bayes factor was calculated as 2 × ΔBIC, and a Bayes factor of greater than 10 indicated strong improvement over the previous model. Lastly, each model was visually inspected to ensure clinically interpretable results.^15–17,27^ Multinomial logistic regression was used to evaluate the association between sociodemographic and clinical characteristics and hydroxyurea adherence trajectory group membership (outcome). All analyses were conducted using SAS 9.4 (Cary, NC), and p<0.05 was considered statistically significant. This study was conducted in accordance with the Strengthening the Reporting of Observational Studies in Epidemiology (STROBE) guideline (Supplemental Table 5).^28^

## Results

### Baseline sociodemographic and clinical characteristics

**Figure 2.**
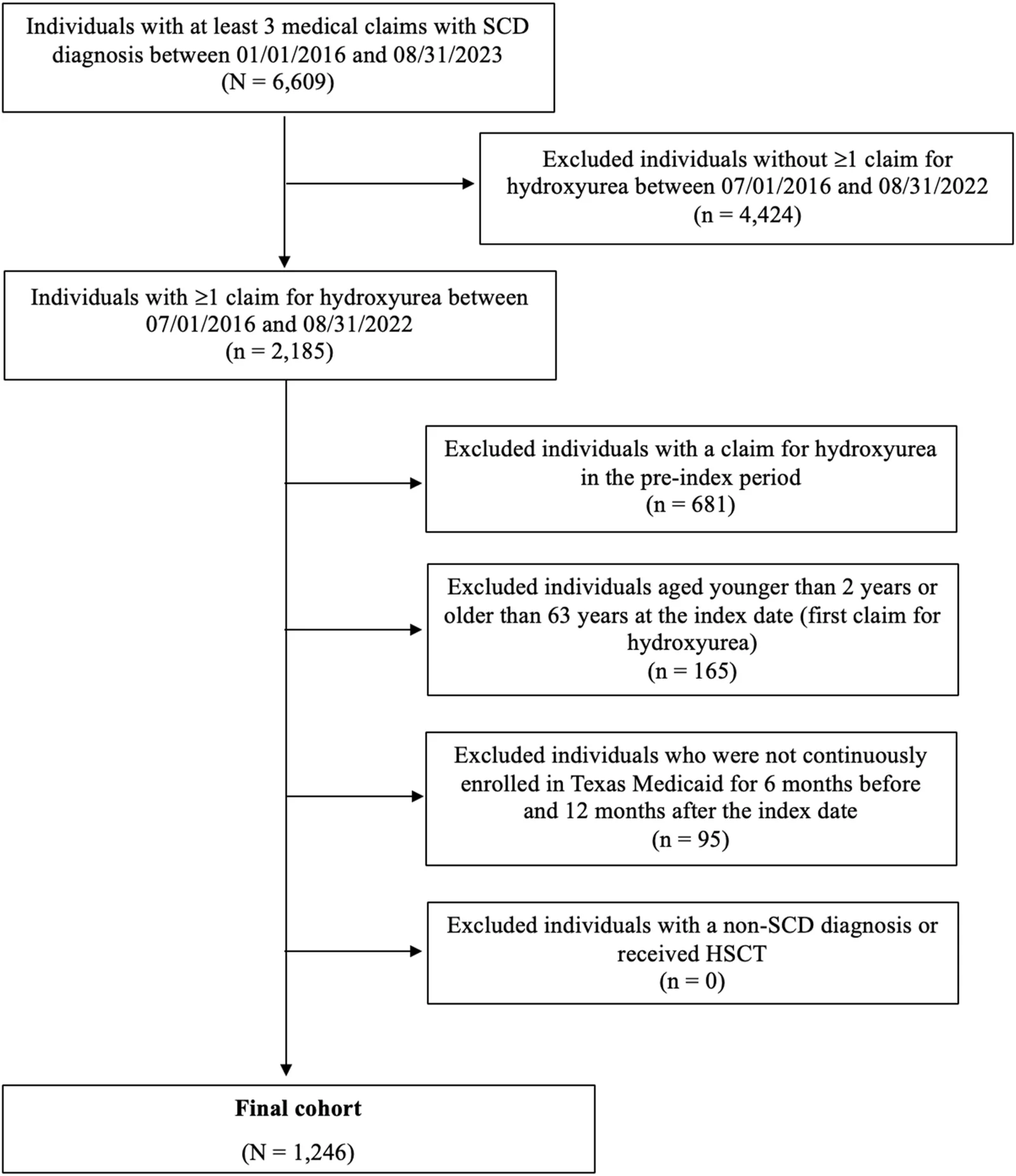
Study attrition. Abbreviation: HSCT = Hematopoietic stem cell transplantation; SCD = Sickle cell disease.

Of the 6,609 individuals with at least 3 inpatient/outpatient claims for SCD during the study period, 1,246 met the eligibility criteria and were included in the analysis (Figure 2). The mean age of the study population was 18.1±12.7, with more than half aged ≤18 years. The proportion of males and females was similar (49.2% vs. 50.8%), and 84.7% resided within <50 miles of an SCD resource. Approximately 70% of individuals with SCD had at least one SCD-related complication, with a mean of 1.7±1.7. Over half (55.4%) had at least one VOC event, with a mean of 1.7±2.5, and 30.7% had at least one other comorbid medical condition, with a mean of 0.4±0.8 during the pre-index period. Additionally, 8.5% had a diagnosis of depression and/or anxiety during the pre-index period (Table 1).

**Table 1.** Sociodemographic and clinical characteristics of individuals with SCD (N = 1,246).

Sociodemographic and Clinical Characteristics	Data (N = 1,246)	
	**n**	**%**
**Sociodemographic Characteristics**		
***Age*** ^a^		
Mean ± SD, years	18.1±12.7	
***Age groups***		
2-15 years	632	50.7
16-21 years	175	14.0
22-34 years	286	23.0
≥35 years	153	12.3
***Gender***		
Male	613	49.2
Female	633	50.8
***Resource availability***		
Lives within <50 miles of an SCD resource	1,055	84.7
Lives within ≥50 miles of an SCD resource	191	15.3
**Clinical Characteristics**		
**Number of SCD-related complications – pre-index**		
Mean ± SD	1.7±1.7	
0	384	30.8
1-2	498	40.0
≥3	364	29.2
***Number of VOC events – pre-index***		
Mean ± SD	1.7±2.5	
0	556	44.6
1-2	405	32.5
3-4	138	11.1
≥5	147	11.8
***Number of other comorbid medical conditions – pre-index***		
Mean ± SD	0.4±0.8	
0	863	69.3
1	265	21.3
≥2	118	9.5
***Depression or Anxiety – pre-index***		
Yes	1,140	8.5
No	106	91.5

Abbreviations: SCD = Sickle cell disease; SD = Standard deviation; VOC = Vaso-occlusive crisis.

^a^Age on index date (first claim for hydroxyurea).

### Adherence to hydroxyurea

**Figure 3.**
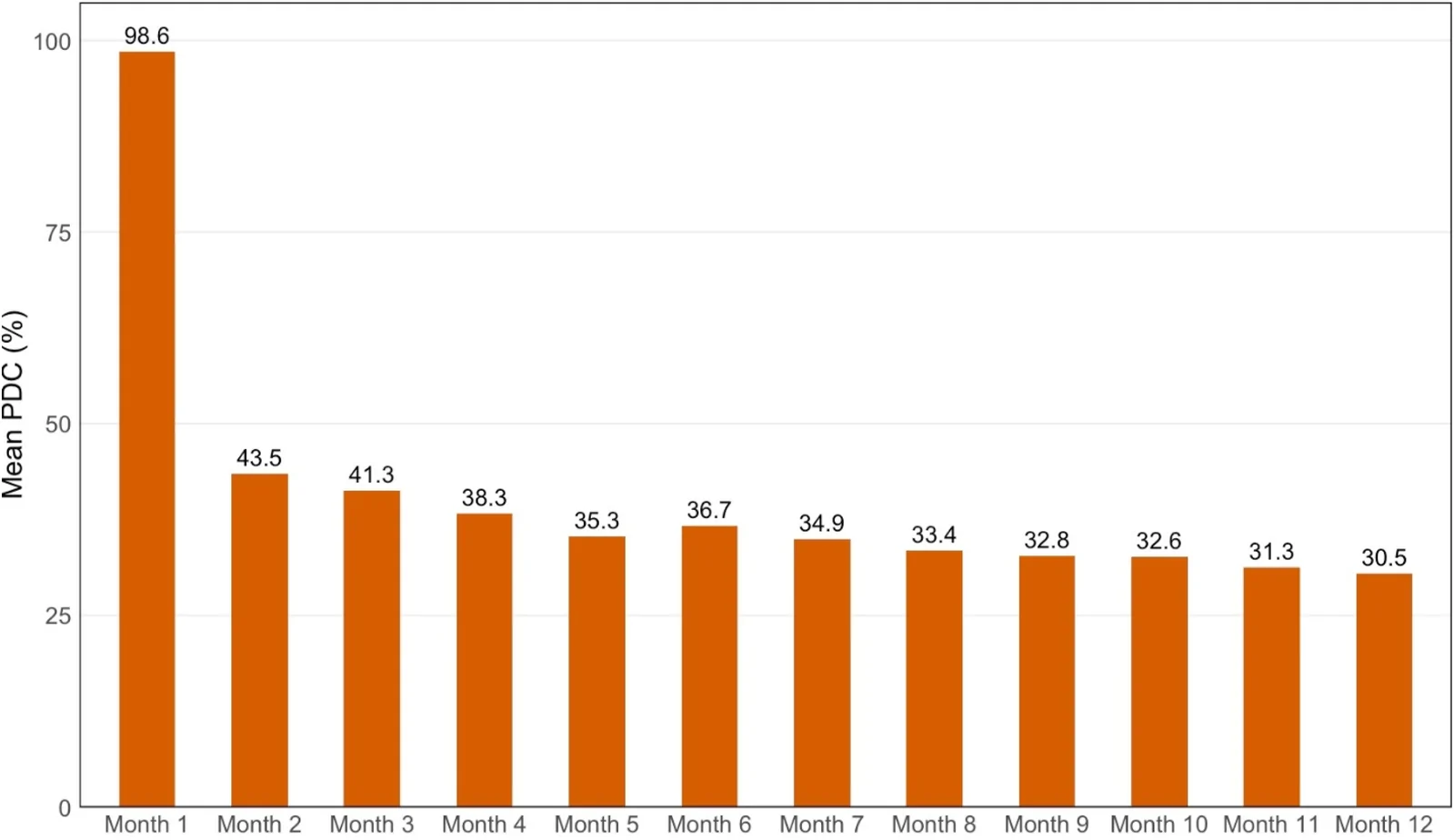
Mean monthly hydroxyurea PDC among individuals with SCD (N = 1,246). Abbreviation: PDC = Proportion of days covered; SCD = Sickle cell disease.

In the first month following hydroxyurea initiation, the mean PDC was 98.6±8.7%, and 97.5% of individuals with SCD were adherent (PDC≥80%). Mean PDC declined to 43.5±44.3% in month 2, with 36.8% meeting the adherence threshold (PDC≥80%). By month 12, the mean PDC was 30.5±40.9%, and 23.4% of individuals with SCD were adherent to hydroxyurea (Figure 3). The overall mean PDC over the 12 months post-index was 40.6±27.8%, with 13.4% considered adherent (PDC≥80%). The overall and monthly hydroxyurea rates (PDC≥80%) can be found in Supplemental Table 3.

### Hydroxyurea adherence trajectories

**Figure 4.**
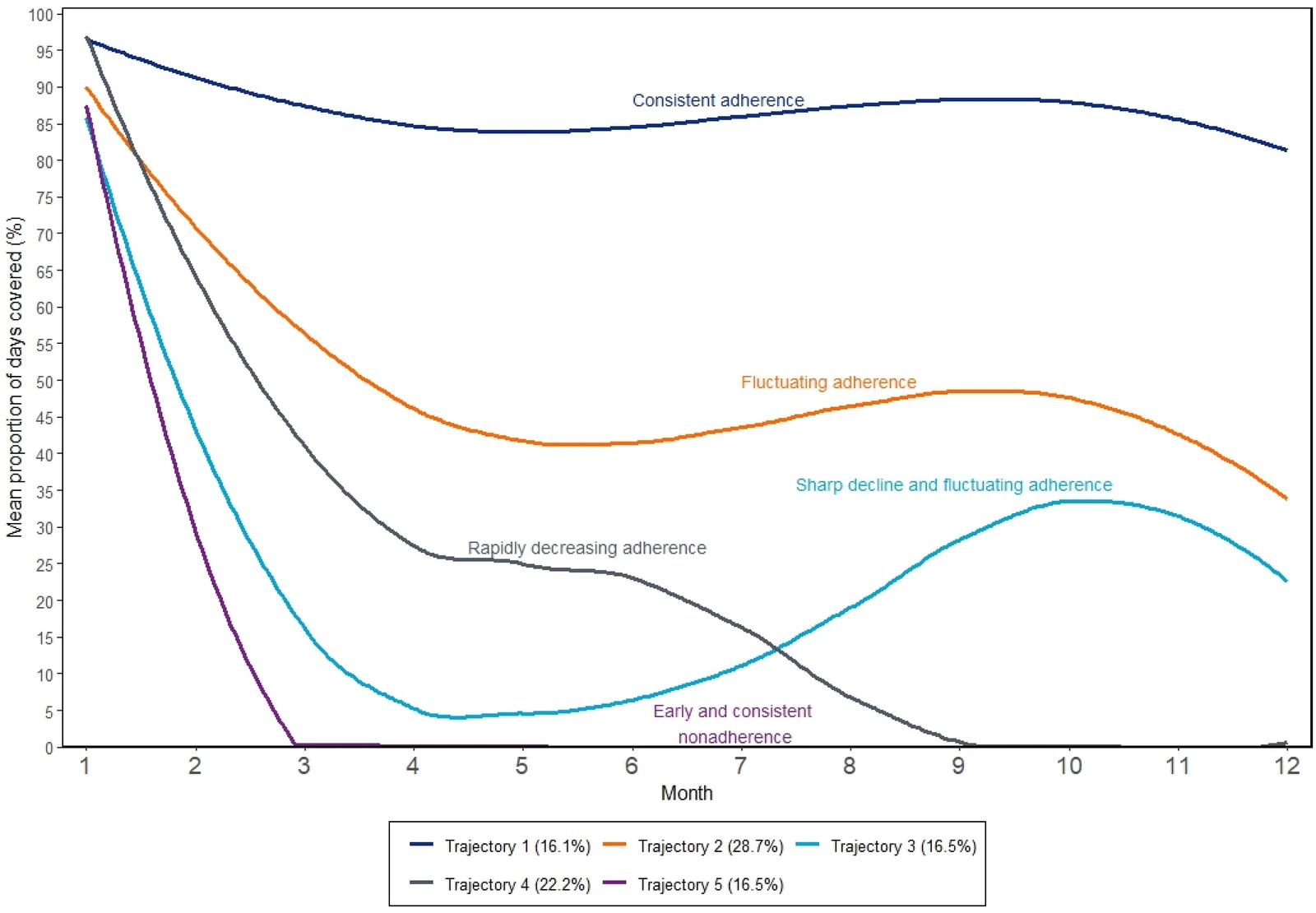
Longitudinal hydroxyurea adherence trajectories among individuals with SCD (N = 1,246). Abbreviation: SCD = Sickle cell disease.

The model with 5 hydroxyurea adherence trajectories had the best fit (Supplemental Table 4) and resulted in the following trajectory groups: 1) Consistent adherence (16.1%; mean PDC = 89.2±8.5%), 2) Fluctuating adherence (28.7%; mean PDC = 52.6±12.8%), 3) Sharp decline and fluctuating adherence (16.5%; mean PDC = 26.2±8.8%), 4) Rapidly decreasing adherence (22.2%; mean PDC = 25.6±10.3%), and 5) Early and consistent nonadherence (16.5%; mean PDC = 8.4±1.3%) (Figure 4). Although the “Sharp decline and fluctuating adherence” and “Rapidly decreasing adherence” groups had similar mean PDC values, their longitudinal patterns differed. The “Sharp decline and fluctuating adherence” group demonstrated an initial decline followed by partial recovery beginning around month 6, whereas the “Rapidly decreasing adherence” group exhibited a continuous decline with no recovery over the follow-up period. Early trough adherence timepoints occurred at months 2, 3, and 5.

The overall multinomial logistic regression model was significant (p<0.0001) and showed that age group and the number of VOC events in the pre-index period were significantly associated with hydroxyurea adherence trajectory group membership (Table 2). Specifically, compared to individuals with SCD aged 2-15 years, those in other age groups (16-21, 22-34, and ≥35 years) had significantly (p<0.05) higher odds of membership (adjusted odds ratio (aOR) range: 2.25-8.72) in the lower adherence trajectories than the consistent adherence trajectory (reference group). Notably, individuals aged 22-34 years had significantly higher odds of membership (aOR range: 2.63-8.72) in each of the lower adherence trajectories, compared with the consistent adherence trajectory. Additionally, an increasing number of VOC events in the pre-index period was significantly associated with higher odds of membership (aOR range: 1.24-1.41) in the lower adherence trajectories compared to the consistent adherence trajectory (Table 2).

**Table 2.** Multinomial logistic regression analysis of factors associated with longitudinal hydroxyurea adherence trajectory group membership (N = 1,246).

Reference: Trajectory 1 Consistent adherence	Trajectory 2: Fluctuating adherence				Trajectory 3: Sharp decline and fluctuating adherence				Trajectory 4: Rapidly decreasing adherence				Trajectory 5: Early and consistent nonadherence			
Independent variables	aOR	95% CI	Wald X^2^	p-value	aOR	95% CI	Wald X^2^	p-value	aOR	95% CI	Wald X^2^	p-value	aOR	95% CI	Wald X^2^	p-value
		Lower				Lower				Lower				Lower		
		Upper				Upper				Upper				Upper		
**Sociodemographic Characteristics**																
**Age groups** ^a^																
16-21 years	1.363	0.788	1.229	0.268	2.245	1.211	6.585	0.010	1.729	0.962	3.351	0.067	1.735	0.898	2.692	0.101
		2.357				4.165				3.106				3.349		
22-34 years	2.627	1.284	6.987	0.008	6.871	3.275	25.982	<0.0001	5.319	2.597	20.868	<0.0001	8.724	4.184	33.387	<0.0001
		5.376				14.417				10.895				18.190		
≥35 years	1.477	0.718	1.123	0.289	3.431	1.605	10.109	0.002	2.755	1.332	7.478	0.006	4.657	2.218	16.527	<0.0001
		3.041				7.338				5.695				9.778		
**Gender**																
Female^b^	0.912	0.640	0.262	0.609	1.104	0.732	0.223	0.637	1.069	0.729	0.116	0.733	1.371	0.906	2.228	0.136
		1.299				1.665				1.566				2.076		
**Resource availability** ^b^																
Lives within ≥50 miles of an SCD resource^b^	1.227	0.749	0.659	0.417	1.428	0.798	1.440	0.230	1.127	0.668	0.201	0.654	0.899	0.520	0.147	0.701
		2.012				2.555				1.901				1.552		
**Clinical Characteristics – pre-index period**																
Number of SCD-related complications	1.031	0.888	0.159	0.690	0.965	0.817	0.176	0.675	0.990	0.846	0.016	0.900	1.126	0.957	2.034	0.154
		1.197				1.140				1.158				1.326		
Number of VOC events	1.239	1.035	5.431	0.020	1.414	1.180	14.107	<0.001	1.400	1.171	13.664	<0.001	1.304	1.087	8.136	0.004
		1.484				1.694				1.673				1.565		
Number of other comorbid medical condition	0.867	0.652	0.962	0.327	0.788	0.578	2.271	0.132	0.770	0.572	2.974	0.085	0.785	0.576	2.343	0.126
		1.153				1.074				1.036				1.070		
Depression or Anxiety^b^	0.844	0.326	0.122	0.728	1.082	0.414	0.026	0.873	1.254	0.497	0.230	0.631	0.682	0.255	0.579	0.447
		2.187				2.825				3.166				1.826		

Abbreviations: aOR = Adjusted odds ratio; CI = Confidence interval; SCD = Sickle cell disease; VOC = Vaso-occlusive crisis.

^a^First claim for hydroxyurea during the study period.

^b^Reference categories: 2-15 years, male, lives within <50 miles of an SCD resource, no diagnoses of depression or anxiety during the 6 months pre-index period.

*Note.* The overall multinomial logistic regression model was significant (p<0.0001).

Odds ratio <1 indicates *lower odds* of membership in the comparison trajectory (i.e., fluctuating adherence, sharp decline, and fluctuating adherence, rapidly decreasing adherence, early and consistent nonadherence) than in the reference trajectory (i.e., consistent adherence); better adherence.

Odds ratio >1 indicates *higher odds* of membership in the comparison trajectory (i.e., fluctuating adherence, sharp decline, and fluctuating adherence, rapidly decreasing adherence, early and consistent nonadherence) than in the reference trajectory (i.e., consistent adherence); lower adherence.

## Discussion

In this longitudinal study of Texas Medicaid beneficiaries with SCD, mean monthly PDC was high at month 1 (98.6±8.7%), with 97.5% considered adherent (PDC≥80%). However, mean monthly PDC declined from 43.5±44.3% in month 2, to 36.7±41.8% in month 6, and 30.5±40.9% in month 12. Of particular concern is the marked decrease from month 1 (98.6±8.7%) to month 2 (43.5±44.3%). The markedly high PDC at month 1 likely reflects the index prescription fill rather than sustained medication-taking behavior, a pattern commonly observed in refill-based adherence measures. The sharp decline observed by month 2 suggests substantial early challenges with adherence following hydroxyurea initiation, highlighting the need for targeted interventions. Overall mean PDC and proportion adherent to hydroxyurea were suboptimal, aligning with prior studies.^5,10,13,14^ Hydroxyurea is recommended as the first-line disease-modifying therapy for eligible individuals with SCD and requires ongoing clinical monitoring, including regular complete blood count (CBC) testing to assess myelosuppression and to guide dose titration.^9^ The need for frequent laboratory monitoring and follow-up visits, particularly during the early phases of treatment initiation and dose adjustment, may present logistic and access-related barriers that contribute to early declines in adherence to hydroxyurea. Similar early declines in adherence have been observed in other chronic therapies requiring ongoing monitoring, such as warfarin and direct oral anticoagulants.^29,30^ In addition, factors such as side effects, perceived lack of clinical benefit, and patient concerns about long-term safety may also influence adherence to hydroxyurea.^31^

After estimating models with 2 to 6 trajectory groups, the 5-trajectory model demonstrated the best fit based on the predefined criteria. Prior SCD studies have measured hydroxyurea adherence cross-sectionally,^10,13,14,32^ and only one prior SCD study has examined longitudinal hydroxyurea adherence trajectories.^18^ Using data from California’s Sickle Cell Data Collection (SCDC) program, Rushing et al. identified 3 hydroxyurea adherence trajectories: “low to no possession” (28%), “moderate to low possession” (35%), and “persistently high possession” (37%) among individuals with SCD enrolled in Medi-Cal.^18^ In contrast, our study identified 5 hydroxyurea adherence trajectories, which may be due to differences in study population (Texas Medicaid vs. California Medi-Cal), study period (2009-2018 vs. 2016-2023), adherence measurement, and model specification (i.e., quadratic vs. cubic polynomials). In the prior study, adherence measurement began at the time of the first refill after the index prescription or after the drug supply days in the index prescription ran out, whichever occurred first,^18^ thereby assessing post-initiation refill behavior rather than adherence following hydroxyurea initiation. Additionally, our study used higher-order (cubic) polynomials, which allow for greater flexibility in capturing nuanced non-linear adherence patterns over time, and used a combination of model fit criteria, consistent with recommended practices for GBTM.^16,17^ These methodological and temporal differences likely contributed to the identification of more granular adherence trajectories in our study. Notably, two trajectory groups identified in our study exhibited similar mean PDC levels during the 12-month post-index period but distinct longitudinal patterns. Specifically, while the “Sharp decline and fluctuating adherence” and “Rapidly decreasing adherence’ groups had comparable mean PDC values, the former showed partial recovery over time, whereas the latter showed a sustained decline without recovery. These distinct patterns may require different intervention strategies; individuals with rapidly decreasing adherence may benefit from early, sustained interventions to prevent persistent nonadherence, whereas those with fluctuating adherence may benefit from ongoing monitoring and reinforcement to maintain improvements.

Our study also identified critical time points for intervention: months 2, 3, and 5 after hydroxyurea initiation. These early timepoints (months 2 and 3) likely correspond to the period of initial prescribing and dose titration.^9^ Interventions implemented upon prescribing and as early as months 2 and 3 may be particularly important in preventing early declines in adherence and setting clear expectations for hydroxyurea therapy. These may also be important time points for healthcare providers to proactively identify and address potential barriers to hydroxyurea adherence, including access barriers such as transportation issues and difficulty obtaining laboratory testing, when possible. Reinforcing education on hydroxyurea, including its benefits, potential side effects, and strategies for managing side effects, may further support continued adherence. Interventions at 5 months after hydroxyurea initiation can re-engage individuals at risk of persistent low adherence who may be experiencing greater barriers to adherence. Considering additional support strategies, such as connecting the individual with an SCD care coordinator, motivational interviewing, and discussing opportunities to switch to other disease-modifying therapies, if possible, may be beneficial. Additionally, individuals with rapidly decreasing adherence and those with early and consistent nonadherence both declined to a mean PDC of 0% at months 9 and 3, respectively. This indicates that these groups of individuals with SCD never refilled their hydroxyurea beyond these time points. Interventions upon initial prescribing such as proactive outreach, including phone calls from the pharmacists or prescheduled follow-up visits with the prescribing provider, may be effective in re-engaging these individuals.

In the multinomial logistic regression, age groups and the number of VOC events in the pre-index period were the factors associated with hydroxyurea adherence trajectory group membership. Young adults aged 16-21 years and adults aged 22-34 and ≥35 had significantly higher odds of membership in the lower adherence trajectories compared to children and adolescents aged 2-15. A similar pattern was observed in Rushing et al.’s study: compared to children and adolescents aged below 17, those aged 18-24 and ≥25 had significantly higher odds (aOR range = 1.70-3.60, p < 0.05) of membership in the lower-adherence hydroxyurea trajectories.^18^ These findings highlight that age group is a driver of longitudinal hydroxyurea adherence patterns and that young adults and adults should be prioritized in receiving adherence interventions. Notably, the age groups 16-21 and 22-34 coincide with the transition period and post-transition from pediatric to adult care, which are well-documented as vulnerable phases in SCD management. During this phase, individuals with SCD often experience disruptions in care continuity, reduced access to adult hematologists, and increased responsibility for self-management,^1,33^ all of which may contribute to suboptimal medication adherence. Hence, implementing targeted interventions for individuals undergoing or who have recently completed the transition to adult care is critical. Equally important is educating adult healthcare providers, as previous studies have noted gaps in providers’ familiarity with SCD management and hydroxyurea use in adult populations.^34,35^ Although we evaluated a broader range of sociodemographic and clinical characteristics than in the previous study,^18^ age groups and the number of VOC events in the pre-index period were significant after adjustment. The lack of significant associations for the other variables may reflect the influence of unmeasured patient-, provider-, or health system-level factors such as patient beliefs, provider communication, and social support, which are not captured in the data used for this study.

Individuals with an increasing number of VOC events in the pre-index period had significantly higher odds of membership in the lower adherence trajectories, suggesting that individuals with more severe disease were less likely to adhere. This pattern may also reflect treatment fatigue among individuals with higher disease burden, as ongoing symptom burden and frequent healthcare utilization may reduce sustained engagement with therapy. Alternatively, individuals who continue to experience recurrent VOC despite hydroxyurea treatment may perceive the medication as ineffective, potentially reducing their motivation to adhere to their treatment. Prior studies have found that individuals with SCD who experience greater disease burden including, higher number of hospitalizations, report more negative perceptions of hydroxyurea, which may lead to nonadherence.^36,37^ Healthcare providers can play a critical role in addressing these perceptions by engaging in open, non-judgmental discussions and shared decision-making with individuals with SCD initiating hydroxyurea. Proactively addressing misconceptions and aligning treatment expectations may help improve adherence to hydroxyurea, particularly among individuals experiencing greater VOC burden. Shared decision-making allows providers and patients to jointly consider the best course of action based on the individual’s disease profile and preferences. Individuals experiencing frequent VOC events may also be experiencing greater barriers to hydroxyurea adherence, which may further contribute to nonadherence. This may also be an opportunity to develop interventions for ED providers when patients present with a VOC. Therefore, individualized and timely interventions to increase hydroxyurea adherence among individuals with SCD experiencing frequent VOC events are needed.

## Limitations

Limitations of this study include the use of prescription claims in estimating adherence, which does not translate to actual usage. However, this method has been used extensively in the literature and is considered reliable.^10,14^ Inpatient hydroxyurea use was not captured, potentially leading to an underestimation of adherence among hospitalized individuals. Additionally, although Texas Medicaid pharmacy claims capture prescriptions reimbursed through Medicaid across dispensing pharmacies, prescriptions obtained outside of Medicaid reimbursement would not be observed and are rare due to no out-of-pocket expenditures with Medicaid. Second, GBTM assumes homogeneity within trajectory groups, which may oversimplify adherence patterns and lead to some misclassification. To mitigate this, multiple model selection criteria were used to ensure robust and meaningful trajectory classification. Third, other important factors which may be associated with hydroxyurea adherence, such as patient-reported factors (e.g., negative perceptions, perceived benefits), side effects, provider counseling, and income,^31,38^ are not available in the Texas Medicaid data and were not included in the analysis. However, although income was not assessed, Medicaid beneficiaries typically include individuals with low-income status.^39^ Fourth, limitations common to administrative claims databases, such as inaccurate coding, may also affect the results of this study. However, miscoding is likely not different between the comparison groups. Fifth, the study period spanned the COVID-19 pandemic, which may have influenced healthcare utilization and hydroxyurea adherence trajectories. Although continuity of care may not have been impacted due to increased telehealth use among individuals with SCD,^40,41^ the impact of the pandemic on hydroxyurea adherence trajectories was not evaluated in this study. Additionally, Texas Medicaid data does not capture clinical reasons for treatment interruption, limiting the ability to distinguish clinically appropriate discontinuation from patient nonadherence. Finally, the generalizability of this study may be limited. However, as over 50% of individuals with SCD are covered by Medicaid and Texas has the third highest prevalence of SCD,^19,20^ findings from this study may be generalizable to a wide range of individuals with SCD.

## Conclusion

Unique adherence trajectory patterns with critical time points for intervention were observed. Individualized and timely interventions should be tailored to young adults and adults with SCD, as well as those experiencing frequent VOC. Future studies should explore the association between patient-reported factors and longitudinal trajectories of hydroxyurea adherence. Studies assessing how adherence interventions impact changes in hydroxyurea adherence trajectories over time are also needed.

## Supplemental material

Supplemental material - Factors associated with longitudinal hydroxyurea adherence trajectories among individuals with sickle cell disease in Texas Medicaid: A group-based trajectory modeling study Supplemental material for Factors associated with longitudinal hydroxyurea adherence trajectories among individuals with sickle cell disease in Texas Medicaid: A group-based trajectory modeling study by Blessing I. Okoye, Godwin Okoye and Jamie C. Barner in Therapeutic Advances in Hematology.

## Data Availability

The source data utilized in this study are not publicly available under the terms of a Data Use Agreement between Texas Health and Human Services and the Health Outcomes Division of The University of Texas at Austin College of Pharmacy.*

## References

[1] Kavanagh PL Fasipe TA Wun T . Sickle cell disease: A Review. JAMA 2022; 328: 57–68. 10.1001/jama.2022.10233 35788790

[2] Hassell KL . Population estimates of sickle cell disease in the U.S. Am J Prev Med 2010; 38: S512–S521. 10.1016/j.amepre.2009.12.022 20331952

[3] Karkoska K McGann PT . Changing trends in sickle cell disease-related mortality in the United States over four decades. Blood 2023; 142: 925. 10.1182/blood-2023-177988

[4] Badawy SM Barrera L Cai S , et al. Association between participants’ characteristics, patient-reported outcomes, and clinical outcomes in youth with sickle cell disease. Biomed Res Int 2018; 2018: 1–8. 10.1155/2018/8296139 PMC6076920 30105252

[5] Okoye BI Barner JC Kang HA , et al. Association between comorbid depression, antidepressant adherence, and disease-modifying therapy adherence among Texas Medicaid patients with sickle cell disease. J Manag Care Spec Pharm 2026; 32: 832–846. 10.18553/jmcp.2026.32.7.832 42341075 PMC13293497

[6] Okoye BI Bhalla A Aiyeolemi AA , et al. Prevalence of mental health conditions among individuals with sickle cell disease in Texas Medicaid. Expert Rev Hematol 2026; 19: 555–562. 10.1080/17474086.2026.2634988 41721824

[7] Udeze C Evans KA Yang Y , et al. Economic and clinical burden of managing sickle cell disease with recurrent vaso-occlusive crises in the United States. Adv Ther 2023; 40: 3543–3558. 10.1007/s12325-023-02545-7 37332020 PMC10329958

[8] Johnson KM Jiao B Ramsey SD , et al. Lifetime medical costs attributable to sickle cell disease among nonelderly individuals with commercial insurance. Blood Adv 2023; 7: 365–374. 10.1182/bloodadvances.2021006281 35575558 PMC9898623

[9] National Heart, Lung, and Blood Institute . Evidence-based management of sickle cell disease: Expert panel report. National Heart, Lung, and Blood Institute, National Institutes of Health, U.S. Department of Health and Human Services, 2014.

[10] Zhou J Han J Nutescu EA , et al. Hydroxycarbamide adherence and cumulative dose associated with hospital readmission in sickle cell disease: a 6-year population-based cohort study. Br J Haematol 2018; 182: 259–270. 10.1111/bjh.15396 29767446 PMC6037608

[11] Quarmyne M-O Dong W Barry V , et al. Hydroxyurea effectiveness in children and adolescents with sickle cell anemia: a large retrospective, population-based cohort study. Blood 2015; 126: 2179. 10.1182/blood.v126.23.2179.2179 27761932 PMC5167640

[12] Charache S Terrin ML Moore RD , et al. Effect of hydroxyurea on the frequency of painful crises in sickle cell anemia. N Engl J Med 1995; 332: 1317–1322. 10.1056/NEJM199505183322001 7715639

[13] Anders DG Tang F Ledneva T , et al. Hydroxyurea use in young children with sickle cell anemia in New York state. Am J Prev Med 2016; 51: S31–S38. 10.1016/j.amepre.2016.01.001 27320463

[14] Kang HA Barner JC Lawson KA , et al. Impact of adherence to hydroxyurea on health outcomes among patients with sickle cell disease. Am J Hematol 2023; 98: 90–101. 10.1002/ajh.26765 36251408

[15] Pennington EL Barner JC Brown CM , et al. Antidepressant adherence using group-based trajectory modeling among postpartum women with Texas Medicaid. J Manag Care Spec Pharm 2025; 31: 167–178. 10.18553/jmcp.2025.31.2.167 39912818 PMC11852796

[16] Nagin DS Odgers CL . Group-based trajectory modeling in clinical research. Annu Rev Clin Psychol 2010; 66: 109–147. 10.1146/annurev.clinpsy.121208.131413 20192788

[17] Nagin DS . Group-based trajectory modeling: an overview. Ann Nutr Metab 2014; 65: 205–210. 10.1159/000360229 25413659

[18] Rushing M Horiuchi S Kayle M , et al. Patterns and predictors of hydroxyurea use among Californians living with sickle cell disease. J Pediatr Hematol Oncol 2025; 47: 169–176. 10.1097/MPH.0000000000003027 40261138

[19] Wilson-Frederick S Hulihan MA Mangum T , et al. Medicaid and CHIP sickle cell disease report, T-MSIS analytic files (TAF). , 2021.

[20] Cells S Mpa EA Jalowsky M , et al. Medicaid access & landscape review for prescription drugs treating sickle cell disease. Sick Cells and Avalere Health, 2022.

[21] Snyder AB Lakshmanan S Hulihan MM , et al. Surveillance for Sickle Cell Disease — Sickle Cell Data Collection Program, Two States, 2004–2018. MMWR Surveillance Summaries; 71. DOI: 10.15585/MMWR.SS7109A1, Epub ahead of print 2025. PMC9552568 36201430

[22] Reeves S Garcia E Kleyn M , et al. Identifying sickle cell disease cases using administrative claims. Acad Pediatr 2014; 14: S61–S67. 10.1016/j.acap.2014.02.008 24882379 PMC7197254

[23] Shah KK Touchette DR Marrs JC . Research and scholarly methods: Measuring medication adherence. J Am Coll Clin Pharm 2023; 6: 416–426. 10.1002/jac5.1771

[24] Odonkor GN Kang HA Barner JC , et al. Medical complications among children and adolescents with sickle cell disease in Texas Medicaid. Healthcare (Switzerland) 2025; 13: 2288. 10.3390/healthcare13182288 PMC12470195 41008420

[25] King K Winkler S Liem R , et al. P1434: The relationship between medication adherence, barriers to medication adherence, and quality of life in sickle cell disease. Hemasphere 2023; 7: e97090b5. 10.1097/01.hs9.0000972620.97090.b5

[26] Rizk JG Qato DM . Transparent reporting of group-based trajectory modeling to study medication adherence: Practical considerations and common pitfalls. J Manag Care Spec Pharm 2026; 32: 507–516. 10.18553/jmcp.2026.32.4.507 41885328 PMC13021367

[27] Jones BL Nagin DS Roeder K . A SAS procedure based on mixture models for estimating developmental trajectories. Sociol Methods Res 2001; 29: 374–393. 10.1177/0049124101029003005

[28] Von EE Altman DG Egger M , et al. The Strengthening the Reporting of Observational Studies in Epidemiology (STROBE) statement: Guidelines for reporting observational studies. Ann Intern Med 2007; 147: 573–577. 10.7326/0003-4819-147-8-200710160-00010 17938396

[29] Mohan A Chen H Deshmukh AA , et al. Group-based trajectory modeling to identify adherence patterns for direct oral anticoagulants in Medicare Beneficiaries with atrial fibrillation. Int J Clin Pharm 2024. 10.21203/rs.3.rs-3938126/v1, Epub ahead of print. 39190225

[30] Kang HR Jones BL Lo-Ciganic WH , et al. Trajectories of adherence to extended treatment with warfarin and risks of recurrent venous thromboembolism and major bleeding. Res Pract Thromb Haemost 2023; 7: 100131. 10.1016/j.rpth.2023.100131 37159747 PMC10163671

[31] Hodges JR Phillips SM Norell S , et al. Intentional and unintentional nonadherence to hydroxyurea among people with sickle cell disease: A qualitative study. Blood Adv 2020; 4: 4463–4473. 10.1182/bloodadvances.2020001701 32941646 PMC7509876

[32] Okoye BI Barner J Kang HA , et al. Association between comorbid depression and antidepressant adherence with adherence to disease-modifying therapies in patients with sickle cell disease in Texas Medicaid. Blood 2025; 146: 6219. 10.1182/blood-2025-6219 PMC13293497 42341075

[33] Saulsberry AC Porter JS Hankins JS . A program of transition to adult care for sickle cell disease. Hematology 2019; 2019: 496–504. 10.1182/hematology.2019000054 31808907 PMC6913425

[34] Lanzkron S Haywood C Hassell KL , et al. Provider barriers to hydroxyurea use in adults with sickle cell disease: a survey of the sickle cell disease adult provider network. J Natl Med Assoc 2008; 100: 968–973. 18717150

[35] Smeltzer MP Howell KE Treadwell M , et al. Identifying barriers to evidence-based care for sickle cell disease: results from the Sickle Cell Disease Implementation Consortium cross-sectional survey of healthcare providers in the USA. BMJ Open; 11. 10.1136/bmjopen-2021-050880, Epub ahead of print 17 November 2021. PMC8601067 34789492

[36] Badawy SM Thompson AA Lai JS , et al. Adherence to hydroxyurea, health-related quality of life domains, and patients’ perceptions of sickle cell disease and hydroxyurea: A cross-sectional study in adolescents and young adults. Health Qual Life Outcomes; 15. 10.1186/s12955-017-0713-x, Epub ahead of print 5 July 2017. PMC5498866 28679417

[37] Haywood C Beach MC Bediako S , et al. Examining the characteristics and beliefs of hydroxyurea users and nonusers among adults with sickle cell disease. Am J Hematol 2011; 86: 85–87. 10.1002/ajh.21883 21117058 PMC3233349

[38] Egiebor IC McCleary KJ Banta JE , et al. Understanding multi-level barriers to medication adherence among adults living with sickle cell disease. Medicine 2023; 102: e35400. 10.1097/md.0000000000035400 37832127 PMC10578734

[39] Medicaid . Eligibility Policy. https://www.medicaid.gov/medicaid/eligibility-policy/index.html, accessed 25 January 2025.

[40] Reeves SL Plegue M Patel PN , et al. Assessing patterns of telehealth use among people with sickle cell disease enrolled in Medicaid during the start of the COVID-19 pandemic. Telemed J E Health 2024; 30: e1971–e1979. 10.1089/tmj.2023.0422 38603584 PMC11257828

[41] Odonkor GN Kang HA Lu A , et al. Telehealth use among Medicaid-enrolled children with sickle cell disease before and during the COVID-19 pandemic. Healthcare (Switzerland) 2025; 13: 1519. 10.3390/healthcare13131519 PMC12249286 40648544

