# Supplementary material for: Factors associated with longitudinal hydroxyurea adherence trajectories among individuals with sickle cell disease in Texas Medicaid: A group-based trajectory modeling study
Source: Ther Adv Hematol. 2026 Aug 24;17:20406207261481884. doi: 10.1177/20406207261481884 (PMC13504119; doi:10.1177/20406207261481884)
Supplement: Supplementary file 1 [file sj-pdf-1-tah-10.1177_20406207261481884.pdf]

SUPPLEMENTARY TABLES

Supplemental Table 1. ICD-10 codes for non-SCD indication and hematopoietic stem cell transplantation

Supplemental Table 2. ICD-10 codes for SCD-related complications, other comorbid medical conditions, depression, and anxiety

Supplementary Table 3. Monthly hydroxyurea adherence rate (PDC≥80%) (N = 1,246)

Supplementary Table 4. Bayesian information criterion, Bayes factor, and predicted trajectory proportions for group-based trajectory models with 2-6 trajectories (N = 1,246)

Supplementary Table 5. Strengthening the Reporting of Observational Studies in Epidemiology (STROBE) checklist for cohort studies

Supplemental Table 1. ICD-10 and CPT codes for non-SCD indication and hematopoietic stem cell transplantation

	**ICD-10 codes**
**NON-SCD INDICATIONS**	
Melanoma	C43, C43.x, C43.xx
Recurrent, metastatic, or inoperable carcinoma of the ovary	C561, C562, C569
Resistant chronic myeloid leukemia	C92.10, C92.12, C92.20, C92.22
Malignant neoplasm of head, face and neck	C76.0
Short bowel syndrome	K90.82
**OTHERS**	**CPT code**
Hematopoietic stem cell transplantation	38240, 38241

Abbreviations: CPT = Current procedural terminology; ICD-10 = International classification of diseases, tenth revision; SCD = Sickle cell disease

Supplemental Table 2. ICD-10 codes for SCD-related complications, other comorbid medical conditions, depression, and anxiety

**SCD-RELATED COMPLICATIONS**	
**Complications**	**ICD-10 codes**
Acute stroke	I63.0-I63.9, I60.0-I60.9, I61.0-I61.9, G45.0-G45.9
Constipation	K59.00-K59.09
Priapism	N48.3, N48.30, N48.32, N48.39
Aplastic episode	D61.89, D61.9
Iron deficiency anemia	D50.9
Cholelithiasis and cholecystitis	K80.0-K80.8, K81.0-K81.9
Hepatic sequestration	K76.89
Intrahepatic cholestasis	K83.1
Osteonecrosis	M87.30-M87.39, M87.80-M87.89, M87.9
Osteomyelitis	M86.00-M86.29
Dactylitis	M79.644, M79.645, M79.646 (pain in fingers)
Vaso-occlusive crisis	D57.0, D57.00, D57.21, D57.219, D57.81, D57.819, D57.41, D57.419
Seizures	G40-G40.9, R56.9
Hyphema	H21.0, H21.0x
Central retinal artery occlusion (Orbital infarction)	H34, H34.x, H34.xx, H43.xxx
Acute chest syndrome	D57.01, D57.211, D57.411, D57.811
Pulmonary embolism	I26.09, I26.99, I27.82
Hematuria	R31, R31.x, R31.xx
Proteinuria	R80. R80.x
Glomerulonephritis	N00.2-5, N00.7, N01.2-5, N01.7, N02.2-5, N02.7, N03.2-5, N03.7, N04.2-5, N04.7, N05.2-5, N05.7, N06.2-5, N06.7, N07.2-5, N07.7,
Acute renal failure	N17.1, N17.2, N17.9
Splenic sequestration	D57.02 (Hb-SS disease with splenic sequestration), D57.212 (Sickle-cell/Hb-C disease with splenic sequestration), D57.412 (Sickle-cell thalassemia with splenic sequestration)
Hypersplenism	D73.1
Cellulitis	L03.x, L03.xx, L03.xxx
Meningitis	A87, A87.x, G00, G00.x, G01, G02, G03, G03.x
Sepsis	A41, A41.x, A41.xx, R65.2, R65.2x
Upper respiratory tract infections	J00, J01, J01.x, J01.xx, J02, J02.x, J03, J03.x, J03.xx, J04, J04.x, J04.xx, J05, J05.x, J05.xx, J06, J06.x
Fever	R50.81
Multisystem organ failure	99592
Congestive heart failure	I50.2, I50.2x, I50.3, I50.3x, I50.4, I50.4x, I50.9
Cardiomegaly	I51.7
Cardiomyopathy	I42, I42x (except I42.6; alcoholic cardiomyopathy, I42.7; cardiomyopathy due to drug and external agent
Dysrhythmia	I49.9

**SCD-RELATED COMPLICATIONS**	
**Complications**	**ICD-10 codes**
Leg ulcers	I83.0, I83.0x, I83.0xx, I83.2, I83.2x, I83.2xx, L97, L97.x, L97.xx, L97.xxx
Stuttering/recurrent Priapism	N48.3, N48.30, N48.32, N48.39
Chronic anemia	D63.1, D63.8, D64.9
Avascular Necrosis	M87.3, M87.3x, M87.3xx, M87.8, M87.8x, M87.8xx, M87.9
Chronic pain syndrome	M54.1, M54.1x, M79.2
Proliferative sickle retinopathy	H36
Pulmonary hypertension	I27.2, I27.21, I29.29, I27.8, I27.89, I27.9
Chronic kidney disease	N17, N17.x, N18, N18.x, N19
Hyposthenuria	403, 404
**OTHER COMORBID MEDICAL CONDITIONS**	
Cardiovascular Disease<br>Coronary Artery Disease<br>Valvular Heart Disease	 I25.10<br>I34.x, I35.x, I36.x, I37.x, I38.x, I39.x
Diabetes Mellitus (Type I & II)	E10.x, E11.x
Hyperlipidemia	E78.0x, E78.1x, E78.2x, E78.4x, E78.5x
Hypertension	I10
Osteoporosis	M80.x, M81.x
Gout	M10.x
Obesity	E66.x
Chronic Obstructive Pulmonary Disease and Bronchiectasis	J40, J41.x, J42, J43.x, J44.x, J47.x
Migraine and chronic headache	G43.x, G44.x
Liver disease, cirrhosis, and other liver conditions	K70.x, K71.0, K71.11, K71.7, K71.8, K71.9, K72.x, K74.x, K75.0, K75.1, K75.8, K75.9, K76.x (except K76.4-peliosis hepatis), K77, K80.3x, K83.0x, R16.0, R16.2, Z48.23, Z94.4
Asthma	J45.x
Epilepsy	G40.x
**MENTAL HEALTH CONDITIONS**	
Anxiety	F400.x, F401, F40298, F408, F409, F41.x
Depression	F32.X, F33.X, F34.1, F06.31, F06.32, F32A, F328, F3289, F338

Abbreviations: ICD-10 = International classification of diseases, tenth revision; SCD = sickle cell disease

Supplementary Table 3. Overall and monthly hydroxyurea adherence rate (PDC≥80%) (N = 1,246)

**Measure**	**PDC≥80**
	**n (%)**
**Month**	
1	1,215 (97.5)
2	459 (36.8)
3	399 (32.0)
4	356 (28.6)
5	329 (26.4)
6	338 (27.1)
7	328 (26.3)
8	320 (25.7)
9	312 (25.0)
10	312 (25.0)
11	300 (24.1)
12	292 (23.4)
**Overall adherence**	
Overall	167 (13.4)

Abbreviations: PDC = Proportion of days covered

Supplementary Table 4. Bayesian information criterion, Bayes factor, and predicted trajectory proportions for group-based trajectory models with 2-6 trajectories (N = 1,246)

**Predicted trajectories proportions**								
**Number of trajectories**	**BIC**	**Bayes factor**	**Trajectory 1**	**Trajectory 2**	**Trajectory 3**	**Trajectory 4**	**Trajectory 5**	**Trajectory 6**
2	-33072.6	-	41.8	58.2	-	-	-	-
3	-32397.0	675.6	25.6	54.8	19.6	-	-	-
4	-32147.1	249.9	18.5	37.0	28.3	16.2	-	-
5	-31885.9	261.2	16.1	28.7	16.5	22.2	16.5	-
6	-31865.0	20.9	17.4	1.9	31.5	17.5	16.9	14.7

Abbreviations: BIC = Bayesian information criterion

Supplementary Table 5. Strengthening the Reporting of Observational Studies in Epidemiology (STROBE) checklist for cohort studies

	Item No	Recommendation	Page No
**Title and abstract**	1	(*a*) Indicate the study's design with a commonly used term in the title or the abstract	2
		(*b*) Provide in the abstract an informative and balanced summary of what was done and what was found	2
**Introduction**			
Background/rationale	2	Explain the scientific background and rationale for the investigation being reported	4, 5
Objectives	3	State specific objectives, including any prespecified hypotheses	5
**Methods**			
Study design	4	Present key elements of study design early in the paper	5
Setting	5	Describe the setting, locations, and relevant dates, including periods of recruitment, exposure, follow-up, and data collection	5, 6, Figure 1
Participants	6	(*a*) Give the eligibility criteria, and the sources and methods of selection of participants. Describe methods of follow-up	5, 6
		(*b*) For matched studies, give matching criteria and number of exposed and unexposed	NA
Variables	7	Clearly define all outcomes, exposures, predictors, potential confounders, and effect modifiers. Give diagnostic criteria, if applicable	6, 7
Data sources/ measurement	8*	For each variable of interest, give sources of data and details of methods of assessment (measurement). Describe comparability of assessment methods if there is more than one group	6, Supplementary Table 1, 2
Bias	9	Describe any efforts to address potential sources of bias	NA
Study size	10	Explain how the study size was arrived at	5, 6, Figure 2
Quantitative variables	11	Explain how quantitative variables were handled in the analyses. If applicable, describe which groupings were chosen and why	6, 7
Statistical methods	12	(*a*) Describe all statistical methods, including those used to control for confounding	7, 8
		(*b*) Describe any methods used to examine subgroups and interactions	7, 8
		(*c*) Explain how missing data were addressed	NA
		(*d*) If applicable, explain how loss to follow-up was addressed	NA
		(*e*) Describe any sensitivity analyses	NA
**Results**			
Participants	13*	(a) Report numbers of individuals at each stage of study—eg numbers potentially eligible, examined for eligibility, confirmed eligible, included in the study, completing follow-up, and analysed	8, Figure 2
		(b) Give reasons for non-participation at each stage	8, Figure 2
		(c) Consider use of a flow diagram	Figure 2
Descriptive data	14*	(a) Give characteristics of study participants (eg demographic, clinical, social) and information on exposures and potential confounders	8, Table 1
		(b) Indicate number of participants with missing data for each variable of interest	NA
		(c) Summarise follow-up time (eg, average and total amount)	8, Table 1
Outcome data	15*	Report numbers of outcome events or summary measures over time	8, 9, Figure 3, Figure 4, Supplementary Table 3
Main results	16	(a) Give unadjusted estimates and, if applicable, confounder-adjusted	9, Table 2

	Item No	Recommendation	Page No
		estimates and their precision (eg, 95% confidence interval). Make clear which confounders were adjusted for and why they were included	
		(b) Report category boundaries when continuous variables were categorized	Table 1
		(c) If relevant, consider translating estimates of relative risk into absolute risk for a meaningful time period	NA
Other analyses	17	Report other analyses done—eg analyses of subgroups and interactions, and sensitivity analyses	NA
Discussion			
Key results	18	Summarise key results with reference to study objectives	9-13
Limitations	19	Discuss limitations of the study, taking into account sources of potential bias or imprecision. Discuss both direction and magnitude of any potential bias	13
Interpretation	20	Give a cautious overall interpretation of results considering objectives, limitations, multiplicity of analyses, results from similar studies, and other relevant evidence	13, 14
Generalisability	21	Discuss the generalisability (external validity) of the study results	13
Other information	22	Give the source of funding and the role of the funders for the present study and, if applicable, for the original study on which the present article is based	15

*Give information separately for exposed and unexposed groups.

Note: An Explanation and Elaboration article discusses each checklist item and gives methodological background and published examples of transparent reporting. The STROBE checklist is best used in conjunction with this article (freely available on the Web sites of PLoS Medicine at http://www.plosmedicine.org/, Annals of Internal Medicine at http://www.annals.org/, and Epidemiology at http://www.epidem.com/). Information on the STROBE Initiative is available at http://www.strobe-statement.org.
